# Supraspinal Circuit Mechanisms Underlying Itch Processing

**DOI:** 10.1002/cns.71022

**Published:** 2026-07-10

**Authors:** Yuncheng Luo, Hao‐Di Tang, Ping Liao, Ruotian Jiang

**Affiliations:** ^1^ Department of Anesthesiology, West China Hospital Sichuan University Chengdu China; ^2^ Laboratory of Anesthesia and Critical Care Medicine, National‐Local Joint Engineering Research Center of Translational Medicine of Anesthesiology, West China Hospital Sichuan University Chengdu China

**Keywords:** brain circuits, contagious itch, emotion, itch, pain

## Abstract

**Background:**

Itch and pain are distinct somatosensory modalities that share overlapping anatomical pathways, particularly in the peripheral nervous system. However, compared with pain, the supraspinal mechanisms underlying itch perception and modulation remain poorly understood, limiting progress in central therapeutic strategies.

**Methods:**

This narrative review synthesizes recent mechanistic studies—primarily from rodent models—to examine supraspinal circuits underlying itch. Relevant literature was identified through targeted database searches and reference tracking.

**Results:**

Accumulating evidence indicates that itch is encoded and modulated by distributed brain networks that contribute to both its sensory perception and emotional components. These networks partially overlap with, yet are distinct from, those mediating pain, revealing convergent and divergent mechanisms at the supraspinal level. Recent studies further demonstrate interactions among itch, pain, and other sensory modalities, highlighting the complexity of central sensory integration.

**Conclusions:**

A clearer understanding of supraspinal itch circuits and their interaction with pain‐related pathways provides important insights into central sensory processing and may facilitate the identification of novel therapeutic targets for chronic and refractory itch.

## Graphical abstract

Peripheral pruriceptive signals ascend from the spinal cord to the brainstem, thalamus, and cortex to encode the sensory features of itch. Parallel projections to limbic and basal ganglia circuits mediate affective and motivational components, including aversion and scratching urge. Descending pathways from cortical and subcortical regions modulate spinal itch transmission and interact with pain circuits. Visual inputs further influence itch through hypothalamic and thalamic networks.

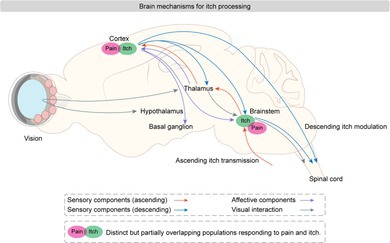

## 1 Introduction

Itch and pain are closely related somatosensory modalities sharing partially overlapping neural pathways yet evoking distinct perceptual and behavioral responses. Chronic itch, a major challenge in dermatological, hepatic, renal, and neurological conditions, is often accompanied by anxiety and emotional distress, underscoring the need to understand central itch processing [1].

Compared with pain, supraspinal mechanisms for itch remain poorly defined, hindering therapy development. Recent work reveals that itch and pain engage partially similar but functionally distinct circuits that interact context‐dependently [2, 3]. Itch is also tightly linked to motivational–affective processes, including the urge to scratch, and can be modulated by multisensory and social cues, highlighting the complexity of central itch regulation [4].

In this narrative review, we synthesize mechanistic studies, primarily from rodent models, to summarize current knowledge of supraspinal circuits underlying itch. We cover ascending and descending sensory pathways, followed by motivational, emotional, and multisensory dimensions. As this review does not follow a predefined systematic protocol, study selection is not exhaustive and may be subject to bias. Pain is discussed when it clarifies shared substrates or modality‐specific mechanisms. Literature was identified through PubMed searches using keywords such as “itch,” “pruritus,” and “neural circuits” with brain region names.

## 2 Brain Areas and Circuits Involved in Itch Sensation

Itch signals are transmitted from the spinal dorsal horn to multiple supraspinal regions, including the brainstem, thalamus, and cortex [5, 6, 7]. Human neuroimaging studies show that itch engages distributed brain networks. Sensory cortices encode the sensory features of itch, motor‐related regions mediate the urge to scratch, and limbic and midbrain structures contribute to the aversive and rewarding dimensions of itch [8, 9, 10, 11, 12, 13, 14, 15, 16, 17, 18, 19, 20, 21, 22]. This section summarizes current knowledge on ascending sensory pathways including cortical coding and the descending modulation of itch. Findings from pain studies are discussed only where they inform shared anatomical substrates or modality‐specific divergence.

### 2.1 Ascending Sensory Pathways of Itch

Following initial processing in the spinal cord, pruriceptive information is conveyed to the brain through two principal ascending pathways: the spinothalamic tract and the spinoparabrachial tract, with spinal projection neurons serving as a critical relay. While the molecular diversity of these projection neurons has been extensively characterized [2, 3, 7, 23, 24, 25, 26], how itch signals are organized and transformed at supraspinal levels remains less understood. Here, we focus on the central components of ascending pathways, emphasizing how parallel thalamic and parabrachial routes distribute itch information to cortical and subcortical targets.

The spinal cord–thalamic pathway constitutes a major route for transmitting itch signals to higher brain centers. Pruritogens activate multiple thalamic nuclei, including the ventral posteromedial (VPM), posterior (Po), anteromedial (AM), ventrobasal complex (VB), central medial nucleus (CM), and mediodorsal thalamus (MD), and inhibition of several of these nuclei attenuates scratching behavior, indicating a facilitating effect on itch [27, 28, 29, 30, 31]. In contrast, the GABAergic thalamic reticular nucleus (TRN) suppresses both itch and pain through projections to VB [30]. Several thalamic outputs project to higher‐order cortical and limbic regions, including AM → anterior cingulate cortex (ACC), MD → ACC, and CM → medial prefrontal cortex (mPFC) pathways, linking sensory relay with affective and motivational processing [28, 31, 32]. Notably, the MD → ACC circuit contains cell type–specific pathways engaged during itch [32]. Under chronic itch conditions, the nucleus reuniens (RE) exhibits increased excitability and contributes to itch maintenance through downstream projections to lateral septum (LS) neurons [33]. Many of these thalamic regions also respond to pain, suggesting a similar supraspinal ascending pathway coding mechanism [27, 30, 34, 35]. Whether distinct thalamic neuronal subtypes selectively encode itch versus pain remains unclear, and some effects on scratching may reflect motor control rather than sensory processing.

In parallel, the spinoparabrachial pathway provides another major ascending route for itch transmission. Glutamatergic neurons in the parabrachial nucleus (PBN) are activated by histaminergic and non‐histaminergic pruritogens, and inhibition of lateral PBN neurons reduces both acute and chronic itch [36, 37, 38].

Within the PBN, functionally distinct neuronal populations contribute to modality‐specific processing. FoxP2^+^ and FoxP2^−^ neurons respectively respond to mechanical and chemical itch and these circuits are recruited under condition of chronic itch [39]. Calcitonin gene‐related peptide (CGRP)‐expressing neurons in the external lateral PBN responded to both pain and itch [40]. Downstream projections from the PBN to limbic structures, including the central amygdala (CeA), further link itch signaling to affective processing, whereas 5‐HT–responsive PBN → CeA circuits appear to selectively modulate itch without affecting pain [31, 41, 42]. Together, these findings suggest that the PBN serves as a critical relay for both itch and pain while containing modality‐selective neuronal populations that may differentially encode pruriceptive and nociceptive information.

Ascending neuromodulatory systems also shape itch processing. The locus coeruleus (LC), the brain's principal noradrenergic nucleus, projects broadly to cortical and limbic regions [43]. Optogenetic activation of the ascending LC–ACC noradrenergic pathway enhances both pain‐ and itch‐related behaviors [44]. However, the contribution of LC signaling appears circuit dependent, as global activation enhances pain without significantly affecting itch [45].

**FIGURE 1:**
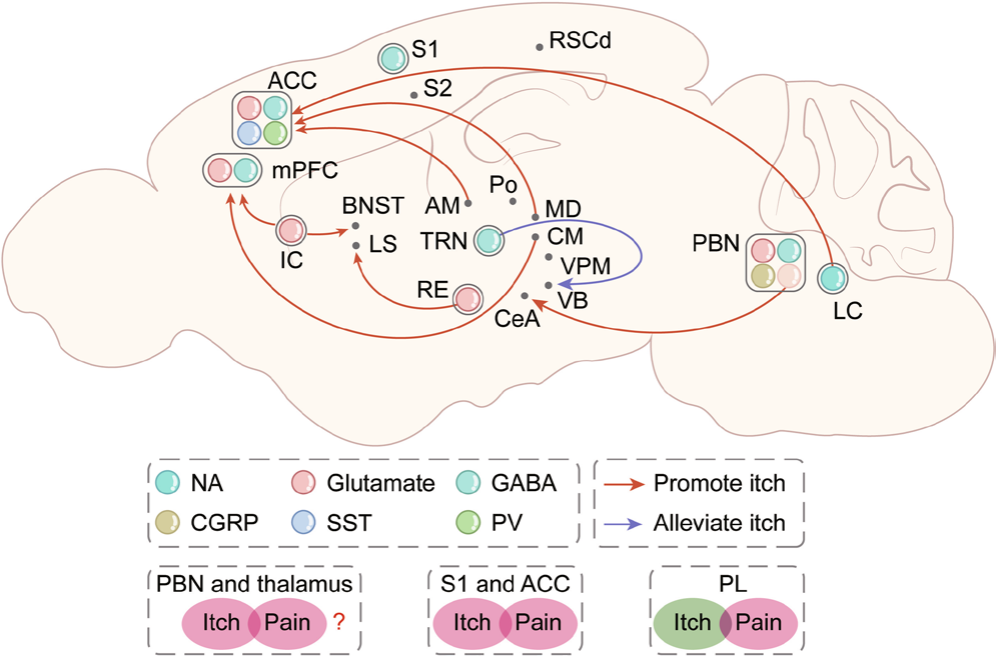
Ascending pathway and cortical processing involved in itch. Schematic representation of major brain regions and neuronal subtypes implicated in itch processing. Multiple cortical, thalamic, brainstem, and limbic regions contribute to the sensory and modulatory components of itch. Red arrows indicate inputs that promote itch, whereas blue arrows indicate inputs that alleviate itch. Circles in the lower represent different neuronal cell types, with the gray solid dot denoting unidentified cell populations. The bottom panels summarize brain regions in which both itch and pain processing have been investigated. Each circle represents neuronal populations involved in itch or pain within the same region. Overlapping areas indicate that the two modalities may recruit partially overlapping neuronal populations. Circle color denotes functional similarity between itch‐ and pain‐related neurons: identical colors indicate similar functional roles in both modalities, while different colors indicate divergent or modality‐specific functions. Question marks indicate that the relationship between itch‐ and pain‐related neuronal populations remains unclear. ACC, anterior cingulate cortex; AM, anteromedial thalamic nucleus; BNST, bed nucleus of the stria terminalis; CeA, central amygdala; CGRP, calcitonin gene‐related peptide–expressing neurons; CM, central medial nucleus; GABA, γ‐aminobutyric acid–expressing neurons; IC, insular cortex; LC, locus coeruleus; LS, lateral septum; MD, mediodorsal thalamus; mPFC, medial prefrontal cortex; NA, noradrenergic neurons; PBN, parabrachial nucleus; Po, posterior thalamic nucleus; PV, parvalbumin‐expressing neurons; RE, nucleus reuniens; RSCd, retrosplenial cortex dorsal part; S1, primary somatosensory cortex; S2, secondary somatosensory cortex; SST, somatostatin expressing neurons; TRN, thalamic reticular nucleus; VB, ventrobasal thalamic complex; VPM, ventral posteromedial thalamic nucleus.

Together, ascending pathways distribute itch signals through parallel but partially overlapping circuits that converge onto cortical and limbic regions, supporting the view that itch processing integrates sensory, affective, and motivational components (Figure 1). However, it remains unclear whether modality‐specific perception arises from distinct neuronal populations, differential activity patterns within shared populations, or engagement of separate circuit architectures. In addition, how different pruritogens are represented and transformed across these ascending pathways remains a major unresolved question.

### 2.2 Itch Coding in Cortex

#### 2.2.1 Somatosensory Cortex

The somatosensory cortex, including the primary (S1) and secondary (S2) regions, constitutes a central node for encoding somatosensory inputs. However, human imaging studies have reported inconsistent involvement of S1 and S2 in itch processing [17, 18, 20, 22, 46], possibly resulting from variability across experimental paradigms and stimulus conditions. Electrophysiological recordings in anesthetized rats have shown that S1 neurons are robustly activated by noxious stimuli and partially responsive to pruritogens, suggesting partially overlapping representations of pain and itch in S1 [47]. More recent in vivo two‐photon calcium imaging in the S1 trunk region (S1Tr) revealed functionally heterogeneous neuronal populations, including multimodal neurons responsive to both itch and pain, as well as itch‐selective neurons [48, 49, 50]. Inhibition of the S1Tr reduces scratching, and itch‐selective neurons exhibit pre‐scratch activation and ignition‐like ensemble dynamics, consistent with a role in itch perception rather than motor feedback [50]. Activity‐dependent labeling further demonstrates partially segregated glutamatergic (pain) and GABAergic (itch) populations in S1 hindlimb cortex, supporting a distributed population‐coding model [49]. However, the role of S2 in itch remains largely unexplored.

#### 2.2.2 Prefrontal Cortex

Beyond sensory representation, the ACC and mPFC integrate sensory, affective, and motivational aspects of itch. ACC pain processing is well characterized at both cellular and circuit levels, with excitatory pyramidal neurons promoting pain hypersensitivity [51, 52, 53]. Pain also induces long‐lasting synaptic plasticity in the ACC, including enhanced potentiation and impaired depression, which may contribute to chronic pain states [51, 53].

Similarly, in chronic itch models, enhanced ACC activity and glutamatergic transmission are observed, and inhibition of glutamatergic neurons reduces scratching, while inhibition of GABAergic neurons exacerbates it [54, 55]. However, cell type–specific studies reveal more complex regulation: activation of glutamatergic neurons suppresses itch, whereas GABAergic neuron activation selectively enhances chemical itch [32]. Among inhibitory subtypes, parvalbumin (PV)‐ and somatostatin (SST)‐expressing interneurons differentially modulate chemical itch, highlighting inhibitory circuit heterogeneity [32]. Regional specialization further shapes ACC involvement in itch. The caudal ACC (cACC) is activated by pruritogens and its inhibition reduces histamine‐induced scratching, whereas rostral ACC (rACC) neurons are suppressed by serotonergic signaling, and global rACC inhibition enhances itch [56, 57, 58, 59]. At the population level, recent evidence demonstrates that itch and pain are represented by distinct neuronal ensembles within layer II/III of the ACC, which receive selective thalamic inputs and differentially regulate behavioral outputs [60].

Within this prefrontal network, the prelimbic (PL) and infralimbic (IL) cortices, key components of the mPFC, also contribute to higher‐order regulation of itch, including sensory evaluation and emotion [61]. PL neurons are activated by pruritogens, and lesion or inhibition consistently reduces scratching [56, 62, 63]. Glutamatergic PL neurons facilitate itch, as shown by activity‐dependent labeling and selective manipulation of itch‐responsive populations [62, 64, 65]. PL neurons receive GABAergic input from the medial septum, in which activation reduces itch [66]. Notably, PL exhibits modality‐specific functional segregation: global inhibition of glutamatergic PL neurons suppresses itch but enhances pain, while itch‐ and pain‐labeled PL neurons form largely distinct, parallel populations that differentially regulate scratching and nociception [64].

In contrast, the contribution of the IL to itch remains less clear. Global inhibition of the IL does not significantly alter itch‐ or pain‐related behaviors [64]. However, pruritogens activate IL glutamatergic neurons, and selective inhibition of these neurons reduces scratching without affecting pain‐related behaviors [67] suggesting functionally specific neuronal subpopulations.

#### 2.2.3 Insular Cortex

The insular cortex (IC) is crucial for processing sensations and emotions. The IC, comprising anterior (AIC) and posterior (PIC) subdivisions, is critically involved in interoceptive processing and the subjective experience of bodily states. Recent studies have extended its role to itch processing. Both AIC and PIC neurons are activated by pruritogens [68]. Inhibition of AIC neurons or AIC projections to the PL and dorsal bed nucleus of the stria terminalis (dBNST) attenuates itch behaviors [69, 70]. Interestingly, inhibition of glutamatergic neurons alleviates both itch and pain, whereas global inhibition affects itch more prominently [69], suggesting differential cell type engagement across modalities. In contrast, manipulating PIC neurons has minimal effects on itch or pain, and PIC activity typically follows scratching onset, suggesting a role in post‐sensory integration rather than primary itch encoding [69].

Beyond primary somatosensory, prefrontal regions and insular cortex, other cortical areas also contribute to itch processing. Activation of glutamatergic neurons in the dysgranular retrosplenial cortex (RSCd) reduces itch responses without affecting pain sensitivity, whereas inhibition of the parietal association cortex alleviates pruritic behaviors, suggesting that distributed associative cortical regions may modulate itch‐related processing [71, 72].

Collectively, cortical itch processing relies on overlapping yet functionally distinct neuronal populations, supporting a distributed coding framework rather than a strict labeled‐line organization (Figure 1). Furthermore, cortical areas such as the ACC and mPFC extend beyond sensory encoding to integrate affective, motivational, and top‐down modulatory components of itch.

### 2.3 Descending Modulatory System of Itch

Top‐down modulation plays a critical role in regulating spinal itch transmission through interactions among cortical, midbrain, and brainstem circuits. Multiple cortical regions exert descending control over itch via projection‐specific pathways. In the somatosensory cortex, glutamatergic S1 neurons projecting to the spinal dorsal horn selectively modulate itch without affecting pain, suggesting a modality‐specific descending pathway [73]. In addition, the parietal association cortex–dorsal striatum circuit also contributes to itch regulation [72].

The ACC exerts top‐down control over itch through projections to the dorsomedial striatum (DMS), ventral tegmental area (VTA), retrosplenial cortex dorsal part (RSCd), and periaqueductal gray (PAG). Glutamatergic ACC → DMS, ACC → VTA, and cACC → RSCd circuits promote scratching behaviors, whereas the rACC → PAG pathway appears to inhibit itch [55, 58, 71, 74]. Interestingly, VTA‐projecting rACC neurons exhibited pruritogen‐dependent modulation of itch [58]. Several of these ACC circuits are also involved in pain facilitation [75, 76, 77, 78].

Similarly, the mPFC, including PL and IL regions, contributes to descending itch modulation through diverse projections. PL → MD and PL → nucleus accumbens (NAc) circuits facilitate itch [65, 79]. More specifically, itch‐responding and pain‐responding glutamatergic PL neurons project to MD and basolateral amygdala (BLA), exhibiting modality‐specific yet opposite modulation on itch and pain [64]. Interestingly, PL → VTA projections exert opposite effects on the two modalities, suppressing itch while facilitating pain [80]. IL excitatory neurons project to the medial striatum, and their inhibition reduces itch responses [67].

The PAG, particularly the ventrolateral PAG (vlPAG), is a major midbrain center for descending modulation of itch [81, 82, 83]. Glutamatergic vlPAG neurons generally facilitate acute and chronic itch, whereas Tac1^+^ glutamatergic neurons selectively regulate itch without affecting pain [81, 82, 83]. In contrast, GABAergic neurons in the vlPAG have been reported to exert opposing effects [81, 82]. Additionally, CB1 receptor deletion in vlPAG GABAergic neurons reduced itch in a dry skin model, while deletion in glutamatergic neurons had no effect [84]. These findings suggest the existence of functionally distinct neuronal subpopulations within the vlPAG that differentially modulate itch processing.

Notably, PAG circuitry exhibits modality‐dependent functional divergence: glutamatergic transmission is generally antinociceptive and GABAergic transmission pronociceptive, suggesting an inverse relationship between pain and itch modulation [81, 82, 83, 85]. However, μ‐opioid receptor activation in the vlPAG suppresses both itch and pain, pointing to shared modulatory mechanisms [86].

The PAG exerts descending control primarily through the PAG–rostral ventromedial medulla (RVM)–spinal axis. Glutamatergic vlPAG → RVM projections induce scratching [81]. Dynorphinergic PAG neurons regulate both itch and pain through κ‐opioid receptor (KOR)‐expressing neurons in the RVM [87]. These KOR^+^ RVM neurons subsequently inhibit spinal itch and pain transmission [88].

The RVM, a key brainstem center for descending somatosensory modulation [89], also regulates itch. For instance, ablation of RVM serotonergic neurons significantly attenuates C48/80‐induced scratching [90]. Classical ON and OFF cells show similar activity patterns in itch and pain paradigms, indicating shared functional organization [91].

However, molecularly defined subpopulations exhibit modality‐specific roles: neurokinin 1 receptor (Tacr1)^+^ and G protein–coupled estrogen receptor (GPER)^+^ ON cells suppress itch while exerting distinct or even opposing effects on pain, whereas KOR‐expressing neurons broadly inhibit both modalities via descending spinal projections [87, 88, 89, 92, 93, 94]. These findings indicate that molecular identity, rather than classical ON/OFF classification, determines modality‐specific output in the RVM [87, 88, 89, 92, 93, 94].

Additional limbic and neuromodulatory circuits also participate in descending itch modulation. CeA → PAG projections facilitate itch [95] whereas CeA pdyn^+^ → PBN circuits suppress both itch and pain [96]. Excitatory BLA → mPFC projections promote itch [97]. The LC also contributes through descending noradrenergic pathways. Inhibition of LC neurons reduces itch but enhances pain. However, conflicting findings across studies may reflect differences in chemogenetic tools (hM4Di vs. PSAM4‐GlyR) and the strategies used to label LC noradrenergic neuronal populations [45, 98].

**FIGURE 2:**
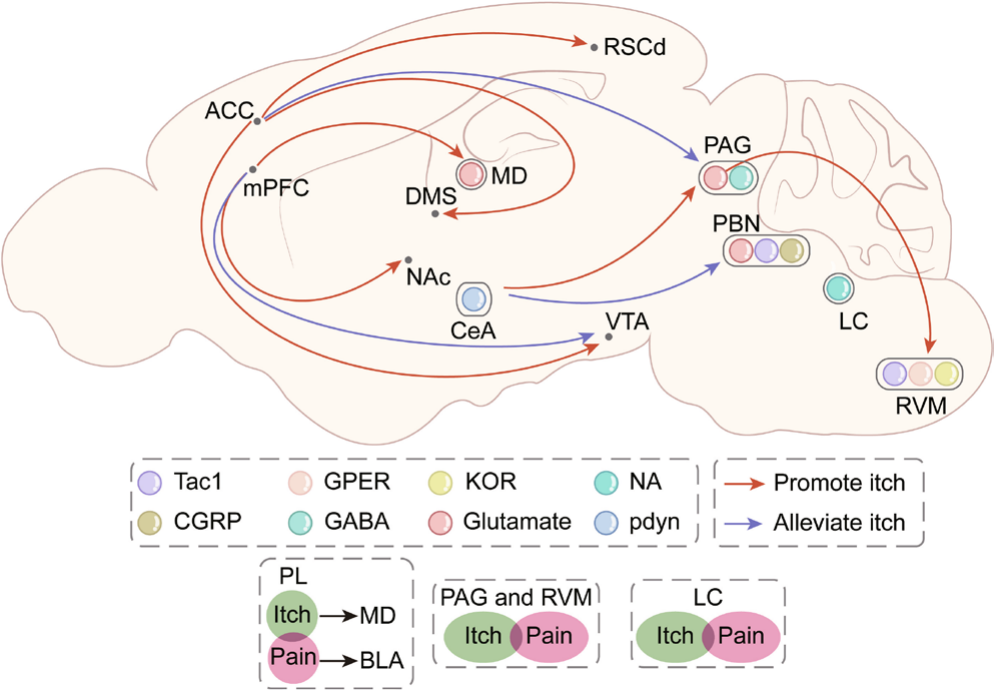
Descending pathway involved in itch processing. Multiple cortical, thalamic, limbic, and brainstem regions contribute to the descending modulation of itch. Red arrows indicate pathways that promote itch, whereas blue arrows indicate pathways that alleviate itch. Colored circles denote distinct neuronal subtypes implicated in itch regulation. The bottom panels summarize brain regions in which both itch and pain processing have been investigated. Each circle represents neuronal populations associated with itch or pain within the same region. Overlapping areas indicate that itch and pain may recruit partially overlapping neuronal populations. Different circle colors denote divergent functions. Arrows in the PL panel indicate distinct downstream projection targets engaged by itch‐ versus pain‐related neuronal populations. ACC, anterior cingulate cortex; BLA, basolateral amygdala; CeA, central amygdala; DMS, dorsomedial striatum; GABA, γ‐aminobutyric acid; GPER, G protein–coupled estrogen receptor; KOR, κ‐opioid receptor; LC, locus coeruleus; MD, mediodorsal thalamus; mPFC, medial prefrontal cortex; NA, noradrenergic neurons; NAc, nucleus accumbens; PAG, periaqueductal gray; PBN, parabrachial nucleus; pdyn, prodynorphin; PL, prelimbic cortex; RSCd, retrosplenial cortex dorsal part; RVM, rostral ventromedial medulla; Tac1, tachykinin 1; VTA, ventral tegmental area.

Collectively, descending modulatory systems form a hierarchical network integrating cortical, limbic, midbrain, and brainstem signals to regulate spinal itch transmission (Figure 2). Although these anatomical circuits substantially overlap with pain pathways, modality‐specific effects emerge from molecularly and functionally distinct neuronal populations, highlighting the complexity of supraspinal itch modulation. Given their causal role in gating sensory transmission, these descending pathways represent promising targets for neuromodulation‐based interventions in chronic itch, including pharmacological and circuit‐based strategies.

Overall, current evidence supports a distributed network in which ascending, cortical, and descending circuits interact to shape itch processing. Ascending pathways, including thalamic and parabrachial circuits, relay pruriceptive signals to the somatosensory cortex for sensory perception and to higher‐order regions such as the ACC and mPFC for affective and motivational processing. Within itch and pain processing, some neurons and pathways preferentially respond to or regulate itch or pain; others are engaged by both modalities, supporting a distributed population‐coding framework rather than a strictly segregated labeled‐line organization. Ascending pathways facilitate both itch and pain, whereas descending systems often exert opposing effects on itch and pain, likely through differential modulation at the spinal level.

However, it remains unclear whether itch and pain are encoded by distinct neuronal populations, shared circuits with different activity patterns, or separate pathways, and whether different pruritogens recruit common or specific mechanisms. In addition, endogenous anti‐itch mechanisms remain less understood than pain modulation. Future studies combining molecularly defined manipulations with real‐time in vivo recordings will be important for dissecting itch‐specific circuit mechanisms and chronic itch‐related plasticity.

## 3 Motivation and Reward of Itch

Chronic itch can cause skin damage from intense scratching motivation, underscoring the need to understand the underlying neural mechanisms. Human neuroimaging implicates a distributed network—including the ACC, motor and premotor cortices, striatum, VTA, and NAc—in motivational and reward‐related aspects of scratching [8, 13, 21]. Consistently, ACC excitatory projections to the DMS and VTA promote itch‐related behaviors, and their inhibition reduces scratching [55, 58, 74].

The VTA plays a central role in reward‐related and goal‐directed behaviors [99]. Both DA and GABAergic VTA neurons are activated by pruritogen‐induced and chronic itch; their activation enhances and inhibition suppresses itch behaviors [55, 100, 101, 102]. Notably, GABAergic neurons exhibit faster activity increases than DA neurons at scratching onset, and functionally, DA neurons regulate scratching recurrence while GABAergic neurons drive the urge to scratch [101]. The VTA receives excitatory inputs from the ACC and PL; inhibiting these pathways reduces itch‐induced aversive and motivational behaviors [55, 58, 80].

Downstream, the NAc, a key motivational hub, shows subregion‐ and cell type–specific contributions, with distinct roles for D1‐ and D2‐expressing neurons. VTA dopaminergic projections to the NAc are activated by itch stimuli [100, 102]. The NAc also receives excitatory PL input, and inhibiting this pathway reduces itch‐related behaviors [65].

Together, these findings frame itch as a motivation‐driven process in which cortical and mesolimbic circuits transform itch signals into relief‐seeking behaviors. The VTA–NAc pathway reinforces the itch–scratch cycle, while cortical inputs (e.g., ACC, prefrontal cortex) shape anticipatory and evaluative aspects. Translating this, chronic itch may involve maladaptive reinforcement of scratching, suggesting that targeting these central mechanisms could disrupt persistent itch–scratch cycles and alleviate symptoms.

Key questions remain, including how itch perception generates the urge to scratch and why itch, rather than pain, preferentially evokes scratching. It is also unclear whether scratch‐induced relief operates similarly in physiological and pathological states. Future studies combining cell‐type‐specific manipulations with real‐time recordings will be essential to dissect the temporal dynamics and causal contributions of these circuits in itch‐driven behavior.

## 4 Emotional and Affective Components of Itch

**FIGURE 3:**
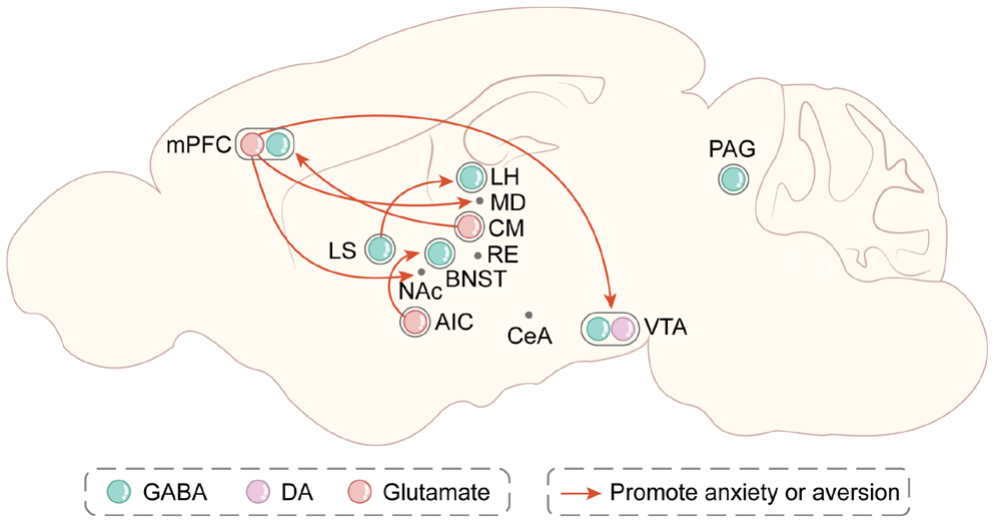
Brain areas and circuits processing emotional component of itch. Schematic diagram illustrating supraspinal circuits implicated in the emotional components of itch. Different brain regions form interconnected networks that regulate aversion and anxiety associated with itch. Circles in the lower left represent different neuronal cell types, with the gray solid dot denoting unidentified cell populations. AIC, anterior insular cortex; BLA, basolateral amygdala; BNST, bed nucleus of the stria terminalis; CeA, central amygdala; CM, central medial nucleus; DA, dopaminergic neurons; GABA, γ‐aminobutyric acid; LH, lateral habenula; LS, lateral septum; MD, mediodorsal thalamus; mPFC, medial prefrontal cortex; NAc, nucleus accumbens; PAG, periaqueductal gray; RE, nucleus reuniens; VTA, ventral tegmental area.

Beyond its sensory‐discriminative features, itch elicits robust emotional responses, including aversion and anxiety. Emerging evidence indicates that these affective components are mediated by distributed yet functionally organized circuits spanning thalamic, cortical, limbic, and midbrain regions, interconnected through projection‐specific pathways (Figure 3).

At the level of thalamocortical and limbic inputs, midline thalamic nuclei play a key role in relaying affective information. The CM sends excitatory projections to the mPFC, driving anxiety‐like behaviors evoked by pruritogens; inhibition of CM or CM–mPFC projections attenuates these responses [31]. In parallel, the reuniens nucleus (RE) projects to GABAergic neurons in the LS, which in turn inhibit GABAergic neurons in the lateral habenula (LH) [33]. Disruption of the RE–LS–LH pathway alleviates itch‐induced anxiety, whereas activation of downstream LH neurons restores aversive affect [33].

Within the mPFC, glutamatergic and GABAergic neurons exert opposing effects on itch‐related emotion. Glutamatergic neurons suppress, whereas GABAergic neurons promote, itch‐induced aversion and anxiety [63]. The PL is a major downstream node mediating these effects. Glutamatergic PL neurons labeled by CQ stimulation promote aversive behavior when optogenetically activated, and their inhibition attenuates conditioned place aversion (CPA) [64]. PL glutamatergic neurons project to both the VTA, MD and NAc, with inhibition of any of these pathways reducing itch‐induced aversion [64, 65, 80]. Notably, pain‐responsive PL neurons instead drive preference, highlighting modality‐specific affective coding within PL circuits [64].

The AIC also contributes to itch‐induced aversion. Glutamatergic AIC neurons project monosynaptically to GABAergic neurons in the dBNST, and inhibition of the AIC–dBNST pathway attenuates CPA [69, 70]. In addition, rACC neurons projecting to VTA GABAergic neurons promote aversive responses, with chemogenetic suppression of this circuit mitigating itch‐induced CPA [58].

At the level of mesolimbic integration, the VTA itself integrates opposing affective signals during the itch–scratch cycle. GABAergic VTA neurons are rapidly activated by pruritogens and mediate the aversive component of itch, whereas dopaminergic neurons are activated following scratching and encode relief and reward [101]. Accordingly, VTA GABAergic neurons drive CPA, while dopaminergic neurons support conditioned place preference [101]. These findings indicate that the VTA integrates negative and positive valence signals to dynamically regulate itch‐related affective states.

The amygdala, a central node in affective processing, plays a critical role in itch. BLA neurons respond to histaminergic and non‐histaminergic itch and are activated in chronic itch states [97, 103]. Enhancing GABAergic tone in the BLA reduces both pain and itch, whereas GABAA receptor blockade selectively enhances itch, and glutamatergic BLA neurons suppress chemical itch [97, 103], pointing to cell‐type‐specific mechanisms. Itch‐activated CeA neurons enhance scratching [95, 104]. CQ‐responsive CeA neurons promote conditioned place aversion and anxiety‐like behavior [95]. Pdyn^+^ CeA neurons attenuate both pain and itch, whereas NPY2R^+^ CeA neurons selectively inhibit itch [96, 105]. However, these CeA subpopulations do not alter anxiety [96, 105], suggesting that other CeA neuronal types mediate negative emotional processing.

In the midbrain, the vlPAG modulates aversion through its GABAergic neurons, which, when inhibited, reduce CPA induced by CQ and chronic itch [82]. In contrast, vlPAG glutamatergic neurons do not appear to contribute significantly to aversive processing [82].

Collectively, these findings support a model in which itch engages distributed affective circuits spanning thalamic, cortical, limbic, and mesolimbic systems to encode negative valence and shape behavioral responses. These networks not only underlie the aversive experience of itch but also contribute to its psychiatric comorbidities, including anxiety and depression. Despite these advances, key questions remain. The causal relationship between itch and negative emotional states is unclear, as chronic itch often coexists with anxiety and depression. How aversive itch signals are dynamically balanced with scratching‐induced reward, and whether emotional changes arise directly from itch‐related circuits or secondarily from altered itch intensity, remain unresolved [102]. Determining whether distinct negative emotions share common or separable neural substrates will be essential for understanding itch‐related affect and its clinical comorbidities. From a translational perspective, targeting affective circuits may therefore provide therapeutic benefit by alleviating both sensory and emotional components of chronic itch.

## 5 Multisensory Modulation of Itch Processing

Beyond unimodal sensory encoding, itch perception is strongly influenced by multisensory and contextual factors. In particular, visual cues and socially transmitted signals can modulate itch‐related responses, reflecting the integration of external sensory inputs with internal representations of somatosensory states. Such phenomena, including contagious itch, highlight that itch is not solely driven by peripheral or spinal inputs, but is dynamically shaped by higher‐order brain processes.

Social mammals are capable of experiencing itch by observing conspecifics. Previous studies have shown that contagious itch is prevalent in humans and involves activation of multiple brain regions, including the AIC, S1, and premotor cortex [4, 106]. Interestingly, this phenomenon has also been observed in mice when they watched conspecifics scratching or viewed scratching behavior in videos [107]. In this process, intrinsically photosensitive retinal ganglion cells (ipRGCs) detect itch‐related visual cues and project to the suprachiasmatic nucleus (SCN) via the retinohypothalamic tract, releasing PACAP and glutamate to activate gastrin releasing peptide (GRP)‐expressing neurons in the SCN that express PAC1 receptors [108]. The GRP–GRPR pathway in the SCN plays a critical role in mediating contagious itch, as optogenetic activation of GRP neurons enhances, while genetic ablation of GRPR‐expressing neurons attenuates this response [107]. Furthermore, GRPR‐expressing neurons in the SCN project to the paraventricular nucleus of the thalamus (PVT), mediating contagious itch [108]. However, the prevalence and behavioral relevance of contagious itch in rodents remain subjects of debate. Similarly, observational pain, the induction of pain‐like responses by witnessing others in pain, has been documented in both humans and rodents [109, 110, 111, 112].

In addition to sensory components, both contagious itch and observational pain are associated with negative emotional states [4, 113]. For instance, contagious itch in mice has been shown to evoke stress‐like responses via a circuit extending from ipRGCs → GRPR‐expressing SCN neurons → PVN (paraventricular nucleus of the hypothalamus) neurons [108]. Additionally, two ventral hippocampal circuits—one projecting to LS GABAergic neurons and the other to dopamine receptor–expressing neurons in the NAc—regulate observational pain‐induced fear [114].

Beyond social interactions, pure visual stimuli, such as light, can also directly modulate pain and itch. Bright light activates ON‐type retinal ganglion cells, which enhance GABAergic activity in the ventral lateral geniculate nucleus/intergeniculate leaflet (vLGN/IGL) [115, 116]. These GABAergic neurons project to the vlPAG and PBN, alleviating pain and suppressing pruritogen‐induced itch, respectively [115, 116]. Green light produces analgesia via a subcortical vLGN → DRN enkephalin circuit and a cortical V2M → ACC circuit that suppresses ACC excitatory activity [117, 118]. In contrast to these established effects on pain, whether green light exerts a similar modulatory effect on itch remains largely unknown.

**FIGURE 4:**
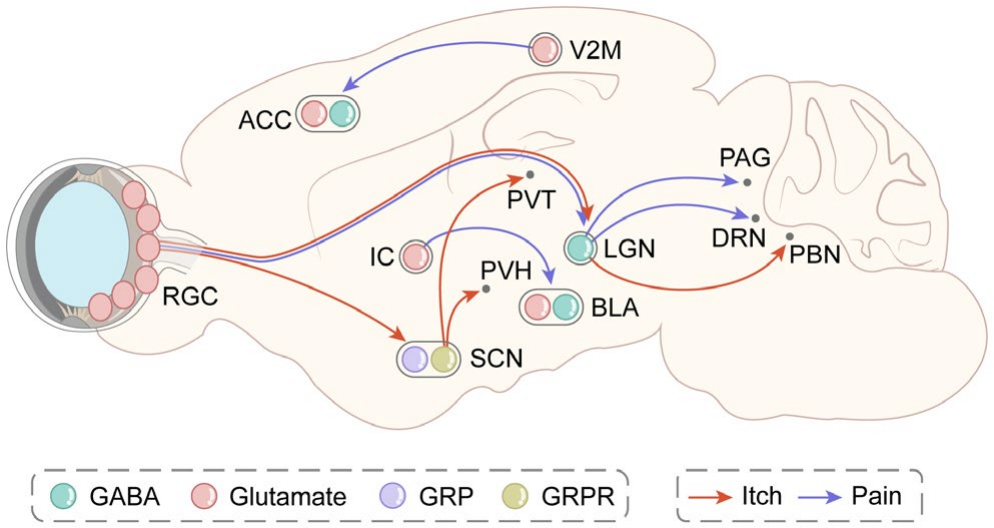
The interaction between itch/pain and vision. Schematic diagram summarizing supraspinal pathways through which retinal ganglion cells (RGCs) transmit visual signals to multiple brain regions implicated in itch and pain regulation. Colored circles indicate distinct molecular neuronal populations engaged in visual modulation of itch (red) and pain (blue) processing. ACC, anterior cingulate cortex; BLA, basolateral amygdala; DRN, dorsal raphe nucleus; GRP, gastrin releasing peptide; GRPR, gastrin releasing peptide receptor; IC, insular cortex; LGN, lateral geniculate nucleus; PAG, periaqueductal gray; PBN, parabrachial nucleus; PVH, paraventricular hypothalamic nucleus; PVT, paraventricular thalamus; RGC, retinal ganglion cell; SCN, suprachiasmatic nucleus; V2M, secondary visual cortex, medial area.

Taken together, these findings demonstrate that multisensory and contextual information—including visual and social cues—dynamically shapes itch processing, influencing not only sensory perception but also associated behavioral and affective responses (Figure 4). However, it remains unclear whether visual modulation of itch, pain, affect, and motivation is mediated by distinct retinal and central circuits or converges on shared pathways, a key unresolved issue in multisensory integration. From a translational perspective, the pronounced influence of multisensory factors suggests that behavioral and environmental interventions could complement pharmacological approaches in chronic itch management. In particular, multimodal strategies incorporating visual or light‐based modulation may enhance the efficacy of conventional therapies.

## 6 Future Perspectives

Recent advances have revealed that itch is processed in the brain through multiple dimensions, including sensory discrimination, motivation, and emotion. However, despite these exciting developments, several key questions remain unresolved.

Most animal models of acute itch rely on chemical pruritogens, which evoke scratching lasting several minutes. In contrast, light touch can trigger scratching through mechanical itch serving to remove irritants, yet the central mechanisms underlying this modality remain poorly understood, and it is unclear whether they share circuit mechanisms with chemical itch. Chronic itch is typically divided into four main categories: (1) dermatological disorders; (2) systemic diseases originating from non‐cutaneous organs; (3) neurological disorders; and (4) psychiatric conditions [119, 120]. Chronic itch is commonly modeled through skin disease paradigms, whereas neuropathic itch—a major clinical challenge—remains difficult to investigate due to the absence of reliable animal models that selectively reproduce itch without altering nociception [121, 122]. Moreover, chronic itch remains a major gap in our understanding, particularly regarding neural plasticity associated with itch–scratch cycles, the development of alloknesis, and the negative emotion. From a translational perspective, identifying neural signatures of subjective itch and developing brain‐ or circuit‐based biomarkers may improve evaluation of therapeutic efficacy. Emerging neuromodulation approaches targeting central circuits, including noninvasive brain stimulation and circuit‐specific pharmacological strategies, also hold promise for treating refractory chronic itch, particularly in patients with prominent affective or motivational symptoms.

Another fundamental challenge in the field lies in the interpretation of behavioral readouts. Scratching behavior is widely used as a proxy for itch in animal studies; however, it reflects a composite output of sensory perception, motor execution, and motivational drive rather than pruriceptive processing itself. This dissociation raises concerns about the interpretability and translational validity of behavioral measures in preclinical models. Moreover, reward‐ and relief‐related circuits may further confound this relationship. Although scratching alleviates itch, it remains unclear whether this effect arises from suppression of aversive sensory signals, engagement of reward/pleasure systems, or both. Addressing these limitations will require refined behavioral paradigms together with in vivo neural recordings to disentangle sensory and motivational components of itch processing.

In addition, sex differences in pain perception are well documented, and some clinical data suggest similar differences in itch [123, 124, 125]; however, the underlying mechanisms remain largely unexplored. Another intriguing phenomenon is the circadian rhythm of chronic itch, yet little is known about the brain circuits that may regulate this daily fluctuation [126, 127]. These questions represent important avenues for future research and may guide the development of more effective, personalized treatments for chronic itch.

## Author Contributions

R.J. performed conceptualization; H.‐D.T. and P.L. performed visualization; R.J. performed supervision; H.‐D.T. and R.J. acquired fundings; Y.L. and H.‐D.T. wrote original draft; R.J. wrote, reviewed, and edited the manuscript.

## Funding

This work was supported by National Natural Science Foundation of China (82571390, 82271249, 32501015), Brain Science and Brain‐Like Intelligence Technology‐National Science and Technology Major Project (2025ZD0214904), 1·3·5 Project for Disciplines of Excellence of West China Hospital of Sichuan University (ZYYC23002), Tianfu Qingcheng Program (ZX2025013), and Sichuan Science and Technology Program (2026NSFSC0974).

## Conflicts of Interest

The authors declare no conflicts of interest.

## Data Availability

Data sharing is not applicable to this article as no datasets were generated or analyzed during this study.
